# The Behavioral and Cognitive Executive Disorders of Stroke: The GREFEX Study

**DOI:** 10.1371/journal.pone.0147602

**Published:** 2016-01-29

**Authors:** Martine Roussel, Olivier Martinaud, Hilde Hénon, Martine Vercelletto, Claire Bindschadler, Pierre-Alain Joseph, Philippe Robert, Pierre Labauge, Olivier Godefroy, on behalf of the GREFEX study group

**Affiliations:** 1 Department of Neurology and Laboratory of Functional Neurosciences EA 4559, SFR CAP-Santé (FED 4231), University Hospital of Amiens, Amiens, France; 2 Department of Neurology, University Hospital of Rouen, Rouen, France; 3 Department of Neurology, University Hospital of Lille, Lille, France; 4 Research Memory Center, University Hospital of Nantes, Nantes, France; 5 Department of Neuropsychology and Neurorehabilitation, University hospital of Lausanne, Lausanne, Switzerland; 6 Research group EA 4136 handicap and nervous system diseases, University of Bordeaux, Bordeaux, France; 7 CoBTeK Research Memory Center CHU, University of Nice Sophia Antipolis, Nice, France; 8 Department of Neurology, University Hospital of Nimes, Nimes, France; University of São Paulo, BRAZIL

## Abstract

**Background:**

Many studies have highlighted the high prevalence of executive disorders in stroke. However, major uncertainties remain due to use of variable and non-validated methods. The objectives of this study were: 1) to characterize the executive disorder profile in stroke using a standardized battery, validated diagnosis criteria of executive disorders and validated framework for the interpretation of neuropsychological data and 2) examine the sensitivity of the harmonization standards protocol proposed by the National Institute of Neurological Disorders and Stroke and Canadian Stroke Network (NINDS-CSN) for the diagnosis of Vascular Cognitive Impairment.

**Methods:**

237 patients (infarct: 57; cerebral hemorrhage: 54; ruptured aneurysm of the anterior communicating artery (ACoA): 80; cerebral venous thrombosis (CVT): 46) were examined by using the GREFEX battery. The patients’ test results were interpreted with a validated framework derived from normative data from 780 controls.

**Results:**

Dysexecutive syndrome was observed in 88 (55.7%; 95%CI: 48–63.4) out of the 156 patients with full cognitive and behavioral data: 40 (45.5%) had combined behavioral and cognitive syndromes, 29 (33%) had a behavioral disorder alone and 19 (21.6%) had a cognitive syndrome alone. The dysexecutive profile was characterized by prominent impairments of initiation and generation in the cognitive domain and by hypoactivity with disinterest and anticipation loss in the behavioral domain. Cognitive impairment was more frequent (p = 0.014) in hemorrhage and behavioral disorders were more frequent (p = 0.004) in infarct and hemorrhage. The harmonization standards protocol underestimated (p = 0.007) executive disorders in CVT or ACoA.

**Conclusions:**

This profile of executive disorders implies that the assessment should include both cognitive tests and a validated inventory for behavioral dysexecutive syndrome. Initial assessment may be performed with a short cognitive battery, such as the harmonization standards protocol. However, administration of a full cognitive battery is required in selected patients.

## Introduction

Executive disorders and action slowing are the most prevalent impairments in stroke patients [1–7] including cerebral hemorrhage [8], cerebral venous thrombosis (CVT) [9] and CADASIL [10]. In the cognitive domain of executive function, rule deduction and shifting are impaired in 20% to 30% of patients, verbal fluency and action speed are impaired in 33% to 60% [1,3,4] and overall impairment is observed in 41% of patients [2]. The few studies of the behavioral domain of executive function have focused on apathy, which was found in 20% to 40% of stroke patients ([11–14]) and 40% of CADASIL patients [15]. The high prevalence of these disorders has prompted the harmonization standards protocol to develop the assessment of executive functions [16, 17]. In addition, executive disorders in stroke-free patients have found to be an independent vascular risk factor [18].

Although these data highlight the frequency of executive disorders in stroke, uncertainties remain that are due to the variable definition and assessment of executive functions across studies. A major confounder is action slowing which has been demonstrated to be more frequently due to sensory-motor disorders than attentional and executive disorder in stroke patients [6] except for postaneurysmal frontal stroke [19]. Another major limitation of previous studies concerns the evaluation of executive functions, which is variable, frequently incomplete and does not control for the impairment of other cognitive functions. In addition assessment of behavioral executive disorders is rarely performed. For example, the Harmonization standards protocol (HSP) [16] for the diagnosis of Vascular Cognitive Impairment has proposed some executive tests (Trail Making Test [20] Fluency test [21]; the Digit Symbol-Coding [22] and a behavioral inventory non-specific of executive impairment. Lastly, the criteria for impairment varied from one study to another and this has been shown to have a major impact on the functions assessed by several performance scores [23]. These observations emphasize the need to use a systematic battery designed according to a theoretical framework defining both behavioral and cognitive aspects of executive functions and controlling for the impairment of instrumental functions. To address these issues, The Groupe de Reflexion pour l’Evaluation des Fonctions EXécutives (GREFEX) has proposed criteria for cognitive and behavioral dysexecutive disorders and a standardized battery [24].

Based on validated diagnosis criteria of dysexecutive disorders, standardized battery [24] and validated framework for the interpretation of neuropsychological data [23], the main objective of our study was to characterize the executive dysfunction profile in stroke; the secondary objectives were (1) to examine the dysexecutive pattern according to stroke subtype (Infarct, hemorrhage and CVT) representative of stroke subtypes referred for cognitive assessment, and (2) to examine the sensitivity of the harmonization standards protocol (HSP) ([16]) using the GREFEX battery as the reference standard [24].

## Methods

### Population

Patients referred for cognitive complaints after stroke were recruited by 11 neurology and rehabilitation centers participating in the GREFEX study.

These inclusions have been performed between 2003 and 2007; at that time a written consent of patients was not required for such research in France, as this study used only routine cognitive test and behavioral interview and consent was obtained orally. Only patients who have given oral consent were included and there is no specific document participant consent. Their participation is voluntary and that they may withdraw from the study at any time. Appropriate oral information of the consent process has been given to the participant including the purpose of the research and expected duration of the subject’s participation (ie, that requires for testing), and the confidentiality of records identifying the patient. These data have been anonymized by using a string built from the first two letters of the patient's last name and from the first letter of the patient's first name. Finally, we confirm that no authors had access to patient identification. Some authors were treating physicians or psychologists. The overall study procedure has been validated by the “Centre d’Evaluation Ethique de la Recherche Non Interventionnelle”, University of Amiens.

The main inclusion criteria were as follows: age between 50 and 90 and a Mini Mental State Examination (MMSE) [25] score ≥16 out of 30. The exclusion criteria were: (1) severe sensorimotor impairment, hemineglect or aphasia precluding cognitive assessment, (2) illiteracy, (3) alcoholism or a severe systemic comorbidity; (4) previous neurologic and psychiatric diseases (other than depression or anxiety), (5) recent introduction of psychoactive or antiepileptic medications and (6) the absence of informed consent. The 237 included patients (Table 1) were assessed following an arterial infarct (n = 57), hemorrhage (n = 54), a subarachnoid hemorrhage (n = 80) and CVT (n = 46). The lesion location was available in 215 patients: 42 had no lesion at imaging (21 ACoA and 21 CVT). In the 173 remaining patients, the lesion was classified as frontal when it included the frontal cortex, posterior when it was restricted to the retrorolandic cortex, hemispheric deep when it spared the cortex and posterior fossa when it was restricted to the cerebellum or the brainstem or both. Lesions (Table 1) were prominent in posterior (n = 67) and frontal (n = 61) regions with a mild right prominence (right-sided: n = 78; left-sided: n = 61; bilateral: n = 34).

**Table 1:** Demographic, imaging and general neuropsychological characteristics of patients.

Stroke population	
**N**	237
**Age**	48.7 ± 15.8
**Gender female:** n (%)	113 (48)
**Education level** 1 / 2 / 3 n (%)	75 (32) / 101 (43) / 61 (26)
**Poststroke delay** (day)	92 ± 112
**Rankin:** 0/1/2/>2 n (%)	68 (31) / 66 (30) / 56 (25) / 32 (14)
**Number of lesion in main locations:** L / R / Bilateral	
**Frontal**	11 / 30 / 20
**Posterior**	37 / 28 / 2
**Hemispheric deep**	11 / 19 / 8
**Posterior fossa**	2 / 1 / 4
**MMSE/Education level 1/2/3**	26.3 ± 3.2 / 27.5 ± 2.5 / 27.7 ± 2.8
**Digit span**	5.5 ± 1.3
**Deficit of oral comprehension:** n (%)	18 (8.5)
**Deficit of oral expression:** n (%)	21 (9.7)
**Deficit of visuo-constructive abilities:** n (%)	30 (14.2)
**Deficit of Episodic memory:** n (%)	71 (33)

Expressed as number and percentage (%) except for age, delay, MMSE score and digit span (mean ± standard deviation)

L: left; R: right; Bilateral: bilateral; MMSE: Mini Mental State

The patients’ performance was analyzed with respect to normative data from the full set of 780 controls in the GREFEX database (mean ± SD age: 48.9±19; female gender: 45%; primary/secondary/higher education: 21%/33%/45%; MMSE: 28.8±1.3; digit span: 5.6±1).

The general neuropsychological assessment varied between centers, as the GREFEX study was conducted according to the routine practice in each center. Assessment of anxiety, depression, language, visuospatial abilities, short-term and episodic memory, and general intellectual efficiency had to be performed using validated tests chosen according to the disease (Table 1). This general cognitive assessment revealed at least one impaired performance in 114 (55.3%) patients.

### Procedures

Dysexecutive disorders were assessed with the GREFEX battery, on the basis of previously validated criteria for dysexecutive disorder and syndrome [24].

#### Executive behavioral inventory

Dysexecutive behavioral changes were assessed using the Behavioral Dysexecutive Syndrome Inventory (BDSI), a structured interview of an informant assessing changes relative to previous behavior in twelve domains: (1) hypoactivity with apathy-abulia; (2) difficulties in anticipation, planning and initiation of activities; (3) disinterest and indifference to his/her own concern and others; (4) hyperactivity-distractibility-psychomotor instability; (5) irritability-impulsivity-aggressiveness; (6) euphoria, emotional lability and moria; (7) stereotyped and perseverative behavior; (8) environmental dependency; (9) anosognosia-anosodiaphoria; (10) spontaneous confabulations; (11) social behavior disorders; and (12) disorders of sexual, eating and urinary behavior [24]. Using a procedure similar to that of the Neuropsychiatric Inventory [26], the informant was first given a screening question that provided an overview of the domain (e.g. for global hypoactivity: “Does the patient have difficulties to spontaneously perform certain activities of daily living, including simple activities that he/she should normally perform without any difficulty”. This could be evidenced by a decrease in spontaneous activities, the need to stimulate the subject to initiate or complete an activity. If the informant provided a positive answer or had a doubt, the domain was then explored with eight questions that provided more detailed information about the specific features of the behavioral disorder. For each disorder with at least one positive answer, the informant had to rate its frequency (1: occasional, less than once a week; 2: sometimes: about once per week; 3: frequent: several days a week but not daily; 4: very frequent, daily) and its severity in everyday life (1: mild; 2: moderate; 3: major). The frequency x severity product was used as behavioral indice. To be interpreted as dysexecutive, behavioral changes (1) could not be more readily explained by perceptuomotor, psychiatric (especially depression, manic state, or obsessive-compulsive disorder), or other cognitive disorders; (2) had to induce significant modifications compared to premorbid behavior; and (3) had to induce significant changes in activities of everyday life, social life, or work.

#### Executive cognitive battery

Cognitive dysexecutive impairments were assessed by using the GREFEX's adaptation of seven tests [24]: the Trail Making Test [20], the Stroop Test [27], the Modified Card Sorting Test [28], a verbal fluency test (naming as many animals and words beginning with letter F in two minutes) [29], the six elements test ([30]), the Brixton Test [31] and a paper and pencil version of the dual task test [32]. The six elements task [30] is a planning test that assesses the ability to time-manage. It consists of three simple tasks (picture naming, arithmetic and dictation) performed twice (thus providing six elements in all), for which subjects have limited time and have to obey switching rule. The score is based on the number of tasks attempted in compliance with the rules. The Brixton test [31] is a rule deduction task in which the participant is presented with a series of cards displaying 10 circles (one of which is filled). The filled circle moves from one card position to another according to rules (n = 9) that have to be found. The score corresponds to the number of errors. In patients for whom time constraints or fatigue precluded the use of the complete battery, investigators were asked to present tests in the following order: verbal fluency, Stroop Test, Trail Making Test, Modified Card Sorting Test, dual task, Brixton task and six elements task. Executive functions were only classified as impaired when they were not more readily explainable by perceptuomotor or other cognitive disorders (language, memory or visuospatial disorders disturbances) [33].

### Statistics

The patients’ performance was analyzed with respect to normative data from the full set of 780 controls in the GREFEX database, according to a validated framework for the interpretation of cognitive data [23].

Briefly, the 19 transformed scores (log transformation for the completion time in the Trail Making and Stroop Tests; a Box-Cox transformation for the other scores) [34] from the seven cognitive tests were fed into a linear regression analysis with age, educational level, gender and interaction term and statistically significant factors in controls were retained. Regression coefficients computed for controls were used to calculate standardized residuals, i.e. z scores (with poor performance corresponding to a negative z score).

To reduce the number of results and as high correlations were observed between some indices, cognitive performance is presented according to 7 executive processes that have been found to be representative of executive deficits reported in the literature: (1) initiation, (2) inhibition, (3) action coordination, (4), information generation (5), rule deduction (6) planning, and (7) flexibility (Table 2). These executive process scores (initiation, inhibition, coordination, generation, deduction, planning and flexibility) were computed from component z scores. The mean value of the seven executive process scores was used as **cognitive summary score.** Scores on the BDSI were also analyzed as z scores. The mean value average of the 12 behavioral z scores was used as the **behavioral summary score** [23].

**Table 2:** Scores in the battery and their combination into seven executive process scores.

Tests	19 scores	Process scores
**Stroop**		
** naming**	Time, Error	Initiation^1^
** reading**	Time, Error	Initiation^1^
** interference**	Time, Error	Inhibition^2^
**Trail Making**		
** part A**	Time, Error	Initiation^1^
** part B**	Time, Error, perseveration	Flexibility^3^
**Verbal fluency** (categorical, letter)	Correct response	Generation^4^
**Modified Card Sorting**	Category	Deduction^5^
	Error, perseveration	Flexibility^3^
**Dual task**	Mu^6^	Coordination^6^
**Brixton**	Error	Deduction^5^
**Six elements**	Rank	Planning^7^

^1^: initiation: average of the z scores corresponding to the completion time (corrected for the error rate in each test) in the Trail Making Test A and Stroop naming and reading subtests;

^2^: inhibition: errors in the interference subtest—errors in the naming subtest of the Stroop Test;

^3^:flexibility: the mean value of the z scores corresponding to perseveration in the Card Sorting and Trail Making Test B;

^4^:generation: the mean value of the z scores corresponding to the fluency tests;

^5^:deduction: of the z scores corresponding to the category achieved in the Card Sorting test and errors in the Brixton test;

^6^: coordination: the z scores corresponding to the mu dual task index; Mu = 100[1-(decrement of digit series recall on dual task + decrement of tracking on dual task)/2]; where dual task decrement of series = proportion of series correctly recalled on single condition—proportion of series correctly recalled on dual condition; dual task decrement of tracking = (number of marked boxes on single condition—number of marked boxes on dual condition)/number of marked boxes on single condition.

^7^: planning: the z scores corresponding to the six elements task.

The dichotomization of performance (normal, impaired) was based on the 5^th^ percentile cutoffs determined from z scores from controls; this approach has been shown to provide, the optimal specificity and sensitivity [23]. The prevalence of cognitive dysexecutive syndrome was determined using the **cognitive summary score** and the prevalence of behavioral syndrome was determined using the **behavioral summary score.**

Effect of stroke subtype on the frequency of dysexecutive syndrome and the executive dysfunction profile has been analyzed using a multinomial logistic regression analysis. Frequency of behavioral and cognitive dysexecutive syndromes was analyzed using 2 multinomial logistic regression analyses with the presence of dysexecutive syndrome as dependent variable (regression 1: presence of behavioral dysexecutive syndrome, regression 2: presence of cognitive dysexecutive syndrome) and the stroke subtype as independent variable (infarct, hemorrhage, ACoA, CVT).

A shortened battery for the diagnosis of behavioral dysexecutive disorders in stroke was determined using a stepwise logistic regression. Regression analyses were performed on z scores from controls and from patients. Control for multicollinearity was achieved by grouping scores on hypoactivity with apathy-abulia with that of difficulties for anticipation and with that of disinterest, referred as global hypoactivity. Regression analyses were performed on the Inventory's 10 component z scores.

The diagnostic accuracy of the (HSP) included executive function, was compared with that of the full cognitive GREFEX battery. This was achieved by comparing the full cognitive GREFEX battery with the scores of the HSP from the GREFEX study (i.e., average of z scores for the Trail Making Test times A and B, perseveration in the Trail Making Test B and both fluency tests). The diagnostic accuracy of these batteries was judged by calculating the area under the curve (AUC) of receiver operating characteristic (ROC) curves, with the corresponding 95% confidence interval (CI), sensitivity, specificity, accuracy, positive predictive value (PPV) and negative predictive value (NPV). A false-positive rate ≤5% (i.e. specificity ≥0.95) was used as a rule of thumb.19 The AUC values were compared by using Delong et al.'s method [35]. The batteries' classifications of patients (as normal vs. impaired) were compared using a McNemar test. This analysis was performed in the study population as a whole and in the subgroup of patients with infarct or hemorrhage.

The relationship with functional outcome was examined using a dichotomized Rankin score (0–2, 3–4) in a stepwise logistic regression with the following 10 factors: age, educational level, sensory-motor impairment, impairment (absent, present) of oral expression, comprehension, visuoconstructive abilities, episodic memory, z score for the MMSE and the cognitive and behavioral dysexecutive summary scores.

Unless otherwise indicated, the threshold for statistical significance was set to p ≤.05. Statistical analyses were performed using SAS 4.3 software (SAS Institute Inc., Cary, NC).

## Results

### Frequency of dysexecutive syndrome and executive dysfunction profile in the overall population

Both the cognitive summary score and the behavioral summary score were impaired in patients (p = 0.0001 for both). The frequency of dysexecutive syndrome was moderate in stroke patients (cognitive syndrome: 39.8%, 95%CI: 33.4–46.3; behavioral syndrome: 44.4%, 95%CI 36.8–52.1). The frequency of combined vs. isolated syndromes was examined in 158 patients for whom both behavioral and cognitive assessments were available. Dysexecutive syndrome was observed in 88 patients (55.7%) (95%CI: 48–63.4): 40 (45.5%) had combined behavioral and cognitive syndromes, 29 (33%) had a behavioral disorder alone and 19 (21.6%) had a cognitive syndrome alone.

All seven executive process scores and behavioral scores (except for social conduct) were impaired. The greatest size effect and high frequencies was observed for initiation and generation in the cognitive domain and hypoactivity and hyperactivity in the behavioral domain (Table 3).

**Table 3:** Cognitive and behavioral executive performance expressed as mean z scores (± Standard Deviation (SD), effect size (d), frequency (%) of impairment and odds ratio (OR).

	Mean SD	*P*	d	Impairment			
				(%)	OR	95%CI	
**Cognitive scores**							
**Overall score**	-0.64±0.92	*0*.*0001*	-0.64	39.8	2.40	1.90	3.03
**Initiation**	-0.90±1.30	*0*.*0001*	-0.90	27.2	2.22	1.71	2.89
**Inhibition**	-0.52±1.69	*0*.*0001*	-0.52	19.5	1.76	1.38	2.26
**Planning**	-0.68±1.54	*0*.*0001*	-0.67	16.3	1.39	1.13	1.72
**Flexibility**	-0.63±1.17	*0*.*0001*	-0.65	20.2	1.81	1.42	2.33
**Deduction**	-0.57±1.66	*0*.*0001*	-0.58	23.9	1.98	1.54	2.56
**Coordination**	-0.21±1.28	*0*.*04*	-0.21	10.6	1.25	1.02	1.53
**Generation**	-0.71±1.20	*0*.*0001*	-0.71	42.9	1.47	1.30	1.66
**Behavioral scores**							
**Overall score**	-1.52±2.71	*0*.*0001*	-1.52	44.4	12.55	7.42	21.3
**Hypoactivity**	-2.22±3.30	*0*.*0001*	-2.22	39.5	13.97	7.7	25.4
**Anticipation**	-1.07±2.03	*0*.*0001*	-1.08	34.6	12.35	7.04	21.7
**Disinterest**	-0.66±1.64	*0*.*0001*	-0.67	28.4	9.27	5.23	16.4
**Euphoria**	-1.29±3.12	*0*.*0001*	-1.29	21.7	7.76	4.16	14.5
**Irritability**	-0.50±1.41	*0*.*0001*	-0.51	14.3	5.93	3.3	10.7
**Hyperactivity**	-1.62±3.86	*0*.*0001*	-1.62	19.8	11.15	5.34	23.3
**Perseveration**	-0.72±2.04	*0*.*0001*	-0.73	22.8	6.23	3.52	11.03
**Dependency**	-8.67±34.71	*0*.*002*	-8.68	6.8	5.5	2.02	15.3
**Anosognosia**	-0.50±1.58	*0*.*002*	-0.51	15.5	9.4	4.59	19.3
**Confabulation**	-11.91±40.10	*0*.*0001*	-11.91	9.3	11.7	3.83	35.8
**Social conduct**	-0.17±1.11	*0*.*2*	-0.18	10.5	7.64	3.1	18.8
**Sexual conduct**	-0.55±2.84	*0*.*02*	-0.55	6.8	5.59	2.03	15.4

### Effect of stroke subtype on the frequency of dysexecutive syndrome and the executive dysfunction profile

**Fig 1:**
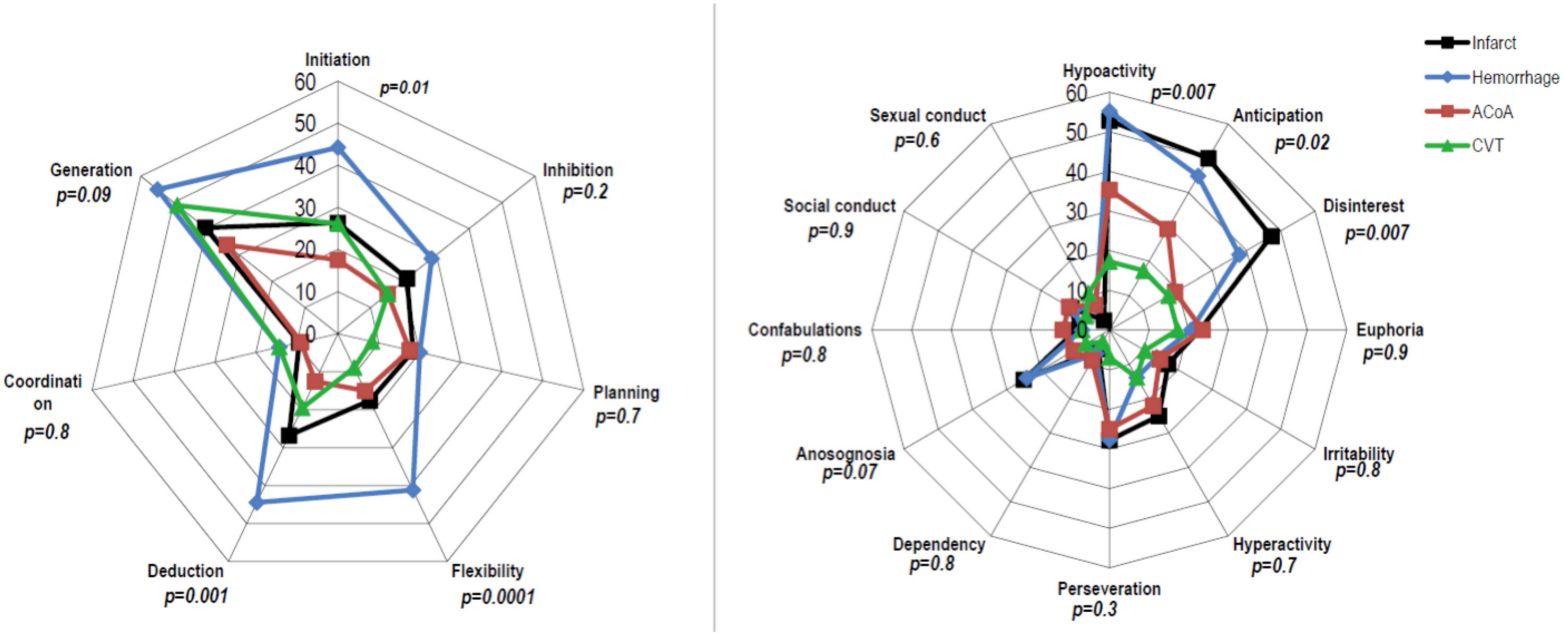
The profile (frequency %) of cognitive (left) and behavioral (right) disorders as a function of the stroke subtype.

The frequency of cognitive dysexecutive syndrome differed (p = 0.001) according to the stroke subtype (infarct: 41%; hemorrhage: 64%; ACoA: 28%; CVT: 37%) due to a higher prevalence in patients with hemorrhage (p = 0.014). The frequency of behavioral dysexecutive syndrome also differed (p = 0.004) according to the stroke subtype (infarct: 56%; hemorrhage: 66%; ACoA: 38%; CVT: 24%) due to a higher prevalence in patients with hemorrhage (p = 0.002) and infarct (p = 0.013). The profile of cognitive impairment in patients with hemorrhage was characterized by higher prevalence of impairment in initiation, flexibility and deduction. The profile of behavioral impairment was characterized by higher prevalence of hypoactivity with disinterest and anticipation loss in patients with infarct and patients with hemorrhage “Fig 1”.

### Establishment and diagnostic accuracy of the shortened inventory for the diagnosis of behavioral executive disorders in stroke patients

The behavioral z scores associated with stroke in logistic regression analysis were hypoactivity (OR: 1.61; 95%CI: 1.27–2.04; p = 0.0001) and hyperactivity (OR: 1.25; 95%CI: 1.01–1.55; p = 0.05).

In a ROC curve analysis, a model with these two behavioral changes provided an AUC of 0.706 (95%CI: 0.644–0.768), which did not differ significantly (p = 0.5) from the value for the full Behavioral battery (AUC = 0.694; 95%CI: 0.631–0.757). The sensitivity was 0.45 (accuracy: 0.64, PPV: 0.95, NPV: 0.51), which did not differ significantly (p = 0.9) from that of the full battery (0.44).

### The GREFEX cognitive battery: comparison with executive tests in the Harmonization Standards Protocol (HSP)

In a ROC curve analysis, both the full cognitive GREFEX battery and the HSP provided very similar (p = 0.27) AUCs (full GREFEX battery: 0.745, 95%CI: 0.704–0.785); HSP: AUC = 0.728, 95%CI: 0.687–0.769). However, the sensitivity of the HSP (sensitivity: 0.32, accuracy: 0.8, PPV: 0.68, NPV: 0.81) was lower (p = 0.01) than that of the full battery (sensitivity: 0.37), since 31 of the 88 (35%) patients classified as impaired by the full cognitive battery were normal when considering the HSP. This lower sensitivity was mainly due to the subgroup of patients with ACoA or CVT. Indeed, 17 of the 37 (46%) ACoA-CVT patients classified as impaired by the full cognitive battery were classified as normal by the HSP (McNemar test: p = 0.007). Conversely the sensitivity of the two batteries did not differ significantly (p = 0.4) in the subgroup of patients with infarct or hemorrhage. (Full GREFEX battery: sensitivity: 0.51, accuracy: 0.88, PPV: 0.52, NPV: 0.93; HSP: sensitivity: 0.46, accuracy: 0.89, PPV: 0.58, NPV: 0.92).

These results indicate that the executive tests from the HSP underestimates cognitive dysexecutive disorders in patients with ACoA or CVT, but not in those with cerebral infarct and hemorrhage.

### Relationship with functional outcome

The logistic regression analysis showed that the following factors were associated with a poor outcome: major sensory-motor impairment (OR: 23.9; 95%CI: 3.01–190; p = 0.003) and the cognitive (OR: 0.119 per 1 SD; 95%CI: 0.04–0.36; p = 0.0001) and behavioral dysexecutive summary scores (OR: 0.719 per 1 SD; 95%CI: 0.56–0.92; p = 0.008).

## Discussion

Our results showed that (1) dysexecutive syndrome was observed in 55.7% (IC 95%:48–63.4) of stroke patients of the GREFEX cohort; (2) the dysexecutive syndrome selectively affected either the behavioral domain or the cognitive domain in more than half of the patients; (3) both behavioral and cognitive disorders were associated with a poor functional outcome; (4) cognitive impairment was more frequent in patients with hemorrhage and behavioral disorders were more frequent in patients with both hemorrhage and infarct; (5) the overall executive dysfunction profile was characterized by prominent impairment of initiation and generation in the cognitive domain and by hypoactivity with disinterest and anticipation loss and hyperactivity in the behavioral domain (6) the selection of executive scores corresponding to that of the HSP resulted in lower sensitivity in patients with ACoA or CVT but not in those with cerebral infarct and hemorrhage.

The present study included patients referred for cognitive complaints and was designed to determine the profile of executive function disorders, as a prerequisite for reliable estimation of the exact prevalence of these disorders in future studies. The finding that a third of stroke patients have a pure behavioral disorder is especially relevant for this purpose, since it indicates that failure to use a specific inventory would entail the omission of a large proportion of impaired patients. Both behavioral and cognitive summary scores are independently associated with a poor functional outcome emphasizing the need to assess both behavioral and cognitive executive functions [24].

The executive dysfunction profiles in the various stroke subtypes were generally similar. In terms of the behavioral profile, the high prevalence of hypoactivity with disinterest and anticipation loss agrees with previous reports of apathy in 20% to 40% of stroke patients [11–14, 36]. The prevalence of the opposite disorder (hyperactive behavior) has not been reported in stroke patients other than those with postaneurysmal frontal lesions [37, 38] and, to a lesser extent, striatal stroke [39, 40]. From a clinical perspective, a shortened inventory involving only two domains (and which takes less than four minutes to administer) provides similar diagnostic accuracy in stroke patients. In terms of the cognitive profile, the high prevalence of a deficit in action initiation suggests that action slowing is a leading post-stroke impairment. Standard cognitive tests cannot reliably distinguish between the two main mechanisms of action slowing (i.e. sensory-motor impairment and initiation of action). This requires specific reaction-time tests, which have shown that action slowing in stroke is mainly due to sensory-motor impairment [6] except in cases of frontal lesions caused by ruptured ACoA aneurysms [19].

The severity of executive function disorders was found to be higher for patients with cerebral hemorrhage and to a lesser extent, infarct. The lower severity of the ACoA and CVT subtypes is likely to be due to the lower prevalence of brain lesions, as already demonstrated for CVT [9] and ruptured ACoA aneurysm [38]. The slightly higher severity of cognitive impairment in patients with cerebral hemorrhage has already been reported [8]; our present results show that this increase concerns executive functions.

The present study had several limitations. The main limitation is the heterogeneity of the population. This study purposefully included patients with various subtypes of stroke in order to analyze the entire range of vascular dysexecutive disorders. Moreover, it included patients referred for cognitive complaints, accounting for the unusual distribution of stroke subtypes with an overrepresentation of CVT and ACoA, which affect relatively young patients. However, our analyses of more prevalent stroke types (i.e., cerebral infarct and hemorrhage) provide much the same findings as for the study population as a whole. This cooperative study has a second limitation. It is a clinical study involving a large number of patients; the complete battery of tests and questionnaires therefore cannot be administered to all patients. A reliable informant was not available in all cases, precluding assessment of behavioral disorders and disability. Despite these limitations, this comprehensive neuropsychological assessment of a large patient population provided unique data on the characteristics of executive function disorders. Our results hint at ways to improve the standardized assessment of post-stroke cognitive impairments. Our comparison with the HSP indicated that it does not underestimate the prevalence of cognitive dysexecutive disorder when it is due to arterial infarct or hemorrhage. From a clinical perspective, our study indicates that the assessment of executive functions in stroke should include both a validated inventory of behavioral dysexecutive syndrome and cognitive tests [41]. In patients with infarct and cerebral hemorrhage, initial assessment may be based on executive function tests from the HSP. However, a full battery of executive tests should then be administered in patients with normal initial assessment and at least one of the following conditions: cognitive complaints, poor functional outcome otherwise unexplained, patients at high risk of cognitive impairment [42] and stroke due to CVT or a ruptured aneurysm.

## Supporting Information

S1 File Executive performance in stroke patients. (XLS)
